# Assessment of gingival translucency at the mandibular incisors with two different probing systems. A cross sectional study

**DOI:** 10.1007/s00784-024-05672-9

**Published:** 2024-06-28

**Authors:** Dimitrios Kloukos, Andrea Roccuzzo, Alexandra Staehli, George Koukos, Anton Sculean, Olga Elpis Kolokitha, Christos Katsaros

**Affiliations:** 1 Department of Orthodontics and Dentofacial Orthopedics, School of Dental Medicine, University of Bern, Freiburgstrasse 7, CH-3010 Bern, Switzerland; 2 Department of Orthodontics and Dentofacial Orthopedics, 251 Hellenic Air Force & VA General Hospital, Athens, Greece; 3 Department of Periodontology, School of Dental Medicine, University of Bern, Bern, Switzerland; 4 Department of Periodontology, 251 Hellenic Air Force & VA General Hospital, Athens, Greece; 5 Department of Orthodontics, Faculty of Dentistry, School of Health Sciences, Aristotle University of Thessaloniki, Thessaloniki, Greece

**Keywords:** Gingival thickness, Periodontal phenotype, Periodontal probe, Colorvue probe

## Abstract

**Objectives:**

Increasing evidence indicates that the thickness of periodontal soft tissues plays an important role in various clinical scenarios, thus pointing to the need of further clinical research in this area. Aim of the present study was to assess gingival thickness at the mandibular incisors by translucency judgement with two different probes and to validate if these methods are comparable and applicable as diagnostic tools.

**Materials and methods:**

A total of 200 participants were included; gingival tissue thickness was measured by judging probe translucency at both central mandibular incisors, mid-facially on the buccal aspect of each tooth using a standard periodontal probe and a set of color-coded probe, each with a different color at the tip, i.e. Colorvue Biotype Probe (CBP). Frequencies and relative frequencies were calculated for probe visibility. Agreement between the standard periodontal probe and the CBP was evaluated via the kappa statistic.

**Results:**

When the periodontal probe was visible, the frequency of CBP being visible was very high. Kappa statistic for the agreement between the standard periodontal probe and the CBP was 0.198 (71.5% agreement; *p*-value < 0.001) for tooth 41 and 0.311 (74.0% agreement; *p*-value < 0.001) for tooth 31, indicating a positive association of the two methods.

**Conclusions:**

An agreement that reached 74% was estimated between the standard periodontal probe and the color-coded probe at central mandibular incisors.

**Clinical relevance:**

In the context of the present study, the two methods of evaluating gingival thickness seem to produce comparable measurements with a substantial agreement. However, in the 1/4 of the cases, the visibility of the color-coded probe could not assist in the categorization of the gingival phenotype.

## Introduction

Variance in gingival anatomy has been in focus of research since the studies of Gargiulo and coworkers who introduced the term “biologic width” and who observed that in corono-apical direction the height of the soft tissue varied not only among individuals but also among teeth [1]. Similarly, a considerable variance was noticed for the tissue thickness. The terms flat/flat-thick and scalloped/scalloped-thin gingival biotype were conceived [2–5]. At the 2017 World Workshop on the classification of periodontal and peri-implant diseases and conditions the “periodontal phenotype” was defined as to describe the three-dimensional volume of the gingiva consisting of i) the gingival thickness (GT), ii) the keratinized tissue width and iii) the width of the buccal bone plate [6–8]. Over the last years the judgement of the phenotype has gained even more attention as it is considered as one of the pillars esthetic outcomes rely on.

In this respect, gingival thickness seems to be one of the factors not only most simply to measure and classify but also showing one of the strongest association with the periodontal phenotype in the literature [8]. According to a recent cone-beam computed tomography (CBCT) scan analysis of 25 healthy patients the labial gingival thickness averaged 1.0 ± 0.3 mm [9] whereby female subjects and canines showed thinner gingiva. Thin gingiva was defined as below 1.0 mm and occurred in 62% of the tested teeth. In general, thin gingival tissue is more prone to gingival recessions than thick gingiva [10, 11]. A recent cross-sectional study found a prevalence of 57.20% for mid-buccal gingival recessions of at least 1 mm on subject and of 14.56% on tooth level in a representative sample of 736 adults living in Turin [12]. When categorizing gingival recessions according to the 2018 classification published by Cairo and coworkers [13] RT 1 recessions mostly affected premolars and maxillary canines whereas RT2 and RT3 recessions mostly maxillary molars and mandibular incisors [12]. With respect to orthodontic tooth movements, in particular proclination of teeth and the prevention of mucogingival defects at lower incisors, careful pretreatment evaluation of gingival thickness should be suggested. The American Academy of Periodontology (AAP) reports that if the dentition moves outside of the alveolar process because of applied orthodontic forces such as arch expansion or thin periodontal phenotypes, a higher incidence of gingival recession and bony dehiscence could be observed [7, 14]. According to a systematic review investigating the evidence on the relationship between periodontal changes caused by orthodontic treatment and gingival phenotype, recession is found inversely related with the gingival thickness. The evidence identified by this systematic review suggested that orthodontic treatment, especially in patients with thin phenotype, might result in periodontal complications; among them gingival recession was the most frequently evaluated [15]. Results of a another recent study revealed a statistically significant decrease in gingival thickness of the maxillary and mandibular anterior teeth after orthodontic treatment with incisor proclination or retroclination [16]. Therefore, it is important to assess gingival thickness before orthodontic tooth movement to preclude potential complications.

For the assessment of gingival thickness several methods have been proposed: visual appraisal of the gingival phenotype, transgingival probing, translucency testing with a periodontal probe, the use of a ultrasonic device (USD) or of cone-beam computed tomography (CBCT) [17]. While some methods might be easy at hand and simple, though less accurate, others might be more precise requiring further cost-intensive infrastructure [17]. Placing of a periodontal probe within the sulcus and evaluating for probe visibility is certainly the most common method for determining the gingival phenotype [18]. Of course, compared to transgingival probing with a probe /acupuncture needle or an USD, probe visibility assessment is not an objective tool since a direct numeric measurement cannot be retrieved. Nevertheless, translucency judgement of the gingiva with a standard periodontal probe for the discrimination between thin and thick gingival phenotype showed a high repeatability and a cut-off level at 0.8 mm [19].

Lately, a new set of colored probes for evaluating visibility has been introduced [11]. There are only few studies that investigated the use of color-coded probes in terms of GT classification. Therefore, the aim of this study was to investigate whether color-coded probes would obtain comparable results to a standard periodontal probe.

## Materials and methods

### Study design

This was a cross-sectional study for which ethical approval was obtained from the Institution`s Ethics and Research Committee (076/7592/06.05.2015). This research was performed in accordance with the Declaration of Helsinki of 1975 and its revised version of Tokyo in 2004.

### Participant enrolment

A total of 200 patients of the Department of Orthodontics and Dentofacial Orthopedics of the 251 Hellenic Air Force Hospital in Athens were consecutively recruited as was previously described [17]. The intended sample size of 200 patients was deemed as appropriate for an adequate powered diagnostic accuracy study, as the current, before study commencement. After obtaining informed consent patients were examined. Inclusion criteria were: presence of all mandibular incisor teeth irrespective of orthodontic therapy or age. Exclusion criteria were: 1) presence of crown restorations or fillings at the cervical part of the anterior mandibular teeth, 2) pregnant or lactating women, 3) presence of clinical signs of gingival conditions/diseases resulting in swelling or color change, or presence of increased probing depth (e.g. > 3 mm), 4) presence of labial recession, 5) intake of medication with any known effect on the periodontal soft tissues, and 6) presence of congenital anomalies or dental structural disorders.

### Clinical parameters

A periodontist (GK) assessed the gingival thickness (GT) and probe visibility at the central mandibular incisors, mid-facially on the buccal aspect of each tooth of each patient. Gingival transparency was judged:

1. With a standard periodontal probe (CPU 15 UNC, Hu-Friedy, Chicago, IL) that was inserted 1 mm deep into the gingival sulcus. A binary classification system was used with the probe either being visible or not visible (Fig. 1).

**Fig. 1:**
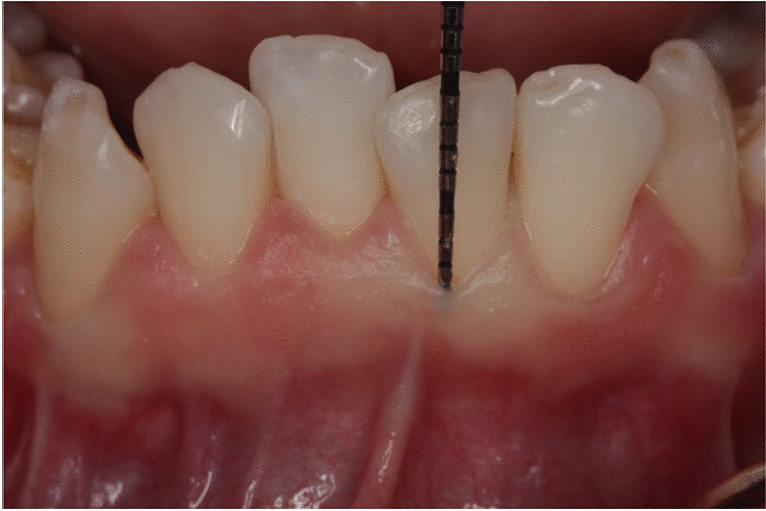
Gingival transparency evaluated with a standard periodontal probe
2. With a color-coded periodontal probe (CBP, Colorvue Biotype Probe, Hu-Friedy, Chicago, IL) with a white, green and blue colored tip was inserted 1 mm deep into the gingival sulcus. The gingival phenotype was then judged based on the visibility of the different colors. Visibility of the white color represented a thin, green a medium and blue a thick gingival phenotype. When none of the colors were visible the phenotype was classified as very thick (Fig. 2).

**Fig. 2:**
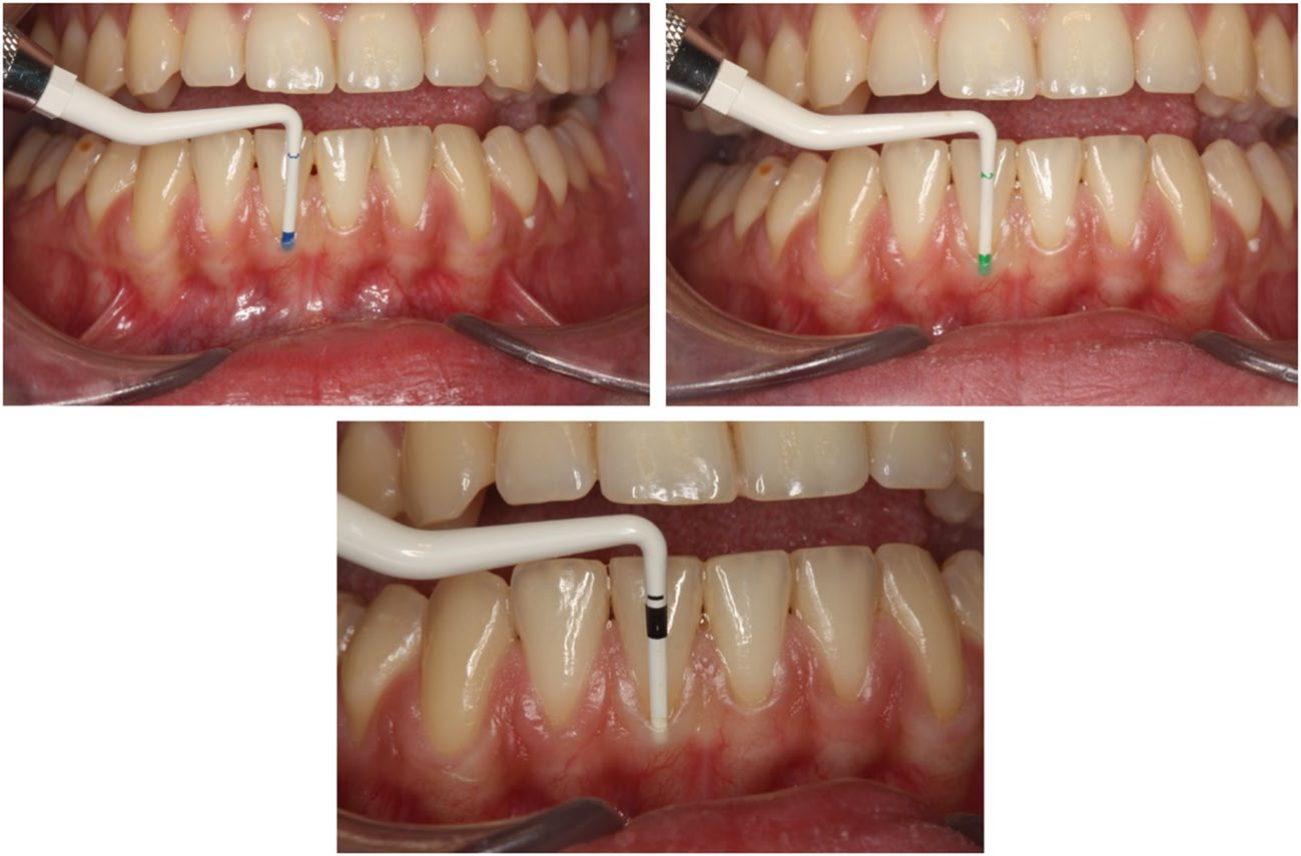
Gingival transparency evaluated with a color-coded periodontal probe

### Intra-examiner reproducibility

The intra-examiner reproducibility of the clinician (GK) who performed all clinical examination was analyzed by examining and re-examining central mandibular incisors of 10 volunteers within two days.

### Statistical analysis

Descriptive statistics (mean, standard deviation, range) were obtained for age while frequencies and relative frequencies were calculated for both probes. Agreement between transparency assessments was evaluated via the kappa statistic and the positive percent agreement (PPA) and negative percent agreement (NPA). All statistical analyses were conducted using Stata 13.0/SE software (StataCorp LP, College Station, TX, USA). Statistical significance was set to α = 5%.

## Results

The mean age of the 200 included patients (104 females, 96 males) was 17.48 ± 7.68 years. Kappa statistic for intra-examiner reproducibility for PB and CBP repeated measurements revealed non significant differences (*p* = 0.022 and *p* = 0.029, respectively). PPA for Tooth #31 was 98.5% (95% CIs: 94.6%, 99.8%) and 97.8% for Tooth #41 (95% CIs: 93.6%, 99.5%). NPA for Tooth #31 was 27.5% (95% CIs: 17.5%, 39.6%) and 18.2% for Tooth #41 (95% CIs: 9.8%, 29.6%).

### Relative frequencies of gingival thickness assessed by a periodontal probe

The evaluation of the measurement is depicted in Table 1. The standard periodontal probe was visible in 67% of teeth #41 and in 65.55% of teeth #31. Not visible was the probe in 33% and 34.5% of the respective teeth.

**Table 1:** Relative frequencies of GT assessed by PB

Frequency (%)		
	Tooth #41	Tooth #31
Visible	134 (67.00%)	131 (65.50%)
Not visible	66 (33.00%)	69 (34.50%)
Total	200 (100.00%)	200 (100.00%)

### Relative frequencies of gingival thickness assessed by the CBP

The results of the color translucency and the classification into gingival phenotype are shown in Table 2. According to transgingival color translucency the gingival tissue was classified into thin, medium, thick, and very thick. For both #41 and #31 thin gingival phenotype was observed in 64% of teeth, medium gingival phenotype in 21.5% (#41) and 21% (#31), thick phenotype in 7% (#41) and 4.5% (#31) and very thick phenotype in 7.5% (#41) and 10.5% (#31).

**Table 2:** Frequencies and relative frequencies (%) for GT assessed by Colorvue

	Tooth #41	Tooth #31
Thin	128 (64.00%)	128 (64.00%)
Medium	43 (21.50%)	42 (21.00%)
Thick	14 (7.00%)	9 (4.50%)
Very thick	15 (7.50%)	21 (10.50%)
Total	200 (100.00%)	200 (100.00%)

When the periodontal probe was visible, the frequency of the visibility of the CBP was very high at both teeth (Tables 3 and 4). The kappa statistic for the agreement between the binary judgement with a periodontal probe and the quadernery judgement with the CBP was 0.198 for tooth #41 which corresponds to 71.5% agreement (*p* < 0.001) and 0.311 corresponding to 74.0% agreement (*p* < 0.001) for tooth #31.

**Table 3:** Relationship between visibility of PB and CBP at tooth #41

	Colorvue at tooth #41				
	Not visible	Visible			
PB/ tooth #41	Very thick (Not visible tip)	Thin (White)	Medium (Green)	Thick (Blue)	Total
Not visible	12(18.18%)	11(16.67%)	30(45.45%)	13(19.70%)	66 (100.00%)
Visible	3 (2.24%)	117(87.31%)	13(9.70%)	1 (0.75%)	134(100.00%)
Total	15(7.50%)	128(64.00%)	43(21.50%)	14(7.00%)	200(100.00%)

**Table 4:** Relationship between visibility of PB and CBP at tooth #31

	Colorvue at tooth #31				
	Not visible	Visible			
PB/ tooth #31	Very thick (Not visible tip)	Thin (White)	Medium (Green)	Thick (Blue)	Total
Not visible	19(27.54%)	18(26.09%)	23(33.33%)	9(13.04%)	69(100.00%)
Visible	2(1.53%)	110(83.97%)	19(14.50%)	0(0.00%)	131(100.00%)
Total	21(10.50%)	128(64.00%)	42(21.00%)	9(4.50%)	200(100.00%)

## Discussion

In this cross-sectional study in a cohort of orthodontic patients, we aimed to investigate soft tissue thickness at the mandibular central incisors by judging translucency of two different periodontal probe systems. We found a positive, albeit weak, correlation between the two measurement techniques. The agreement of the two methods was 71.5% for tooth #41 and 74.0% for tooth #31.

When comparing these results with those reported previously, it seems that these two methods do not produce consistent measurements with absolute agreement. Bertl and coworkers determined GT at maxillary anterior teeth and on photographs depicting merely the gingiva with the inserted probes. They then compared these evaluations with transgingival sounding with an endodontic file. In terms of intra-examiner repeatability and inter-examiner reproducibility [20] both probes showed a high variability, though the colored probes performed worse. With them most cases were classified as “medium” and the colored probes failed to detect GT ≤ 1 mm in 88% of the cases [20]. The repeatability of a CBP has previously been tested as low [17].

Gingival thickness evaluation by probe visibility/invisibility has been widely used since it was first described in 2003 by Kan et al. [21] and still is controversially discussed. Our group reported on a cutoff of 0.8 mm while Frost et al. failed to determine a GT threshold that can reliably discriminate between sites where the probe was visible and where it was not [22]. The GT that most closely could be related with probe invisibility was > 0.8 mm [22]. Nevertheless, high reproducibility with 85% agreement between duplicate measurements have been reported [18].

An interesting investigation was made by Aslan et al. who determined thickness cutoffs for phenotype probing using colored probes and correlated those with soft tissue thickness measurements made on CBCT scans. According to color visibility the phenotype was classified as thin, medium, thick, or very thick and the respective cutoffs on the CBCT were 0.83 mm between thin and medium, 1.07 mm between medium and thick, and 1.24 mm between thick and very thick phenotype. Interestingly, the correlation of CBCT measurements and probe classification was higher when only maxillary anterior teeth were considered [23].

When one compares different methods, it becomes clear that results substantially vary among different methods and so far, there is no accurate, reliable, yet simple measurement method broadly accepted among researchers all over the world. Transgingival probing, which is an invasive method, mostly depends on the infusion of the anaesthetic agent, the angulation of the probe, the instrument, or the distortion of tissues. Ultrasound measurement require experience and can be distorted by the directionality of the tip. Measurements made by ultrasonography were found to exceed transgingival probing with a periodontal probe by 0.16 mm [17]. No differences were reported between a digital puncture method and ultrasound in a cohort of Indian adults [24]. In another Indian population, a significant discrepancy between transgingival puncture and ultrasound was found [25], interestingly, with a lower accuracy for the ultrasound. Another non-invasive method that has been described is CBCT. Although by CBCT distances can be easily measured, similar radiographic densities of soft tissues like lips, cheeks, tongue and gingiva might conceal the identification of the gingiva. In some cases gingival tissues are even too thin to be measured. Other studies have introduced several approaches for comprehensive visualization and quantification of oral soft tissue thickness utilizing fusion of optical 3D and CBCT images or by implementing an altered method of CBCT image acquisition. These procedures have demonstrated efficiency in measurement abut have also introduced an added workflow complexity [26, 27].

Comparing CBCT with ultrasound, CBCT resulted in higher values of about 0.13 mm to 0.21 mm as USD [28]. Concerning reproducibility both CBCT and ultrasound evaluation of GT proved to be reliable [28]. In a Chinese cohort of young healthy adults excellent consistency was obtained between transgingival probing and CBCT evaluation whereas inconsistent results were found between transgingival probing/CBCT and probe transparency [29].

Different findings among studies might be explained by the fact that not all methods were used on the same sample of patients. Then not all tooth types of the same cohort were examined, while some studies focused on maxillary anterior teeth this study investigated GT at mandibular central incisors. Furthermore, the respective methods and study settings vary considerably. For example, Bertl et al. evaluated probe transparency on photographs where the probes outside the gingival tissue was covered so that only the tip within the gingival tissue could be judged [20]. Here the probe visibility was judged clinically.

When interpreting the present findings, it is important to note, that this study did not include different ethnicities and subjects with different degrees of gingival tissue pigmentation. Thus, it cannot be ruled out that ethnicity and degree of gingival pigmentation might impact gingival translucency, which in turn might represent a limitation of this study. Furthermore, one has to be aware of the fact that only central lower incisors of young orthodontic patients were considered, and it is well known that tooth type, position in the arch as well as age appear to have an impact on gingival thickness as well. As final remark, it must be mentioned that finding a precise and consistent measurement method for GT might be a difficult task to tackle since all clinical or imaging methods may hold an inherent measurement error.

## Conclusions

Our results showed an agreement that reached 74% between the standard periodontal probe and the color-coded probe at the mandibular incisors. In the context of the present study, the two methods of evaluating gingival thickness seem to produce comparable measurements with a substantial agreement. However, in the 1/4 of the cases, the visibility of the color-coded probe could not assist in the categorization of the gingival phenotype.
